# Data on functional characterization of LECT2 from *Lampetra japonica*

**DOI:** 10.1016/j.dib.2018.02.036

**Published:** 2018-02-15

**Authors:** Zhiliang Wang, Jiali Lu, Qingwei Li, Yue Pang

**Affiliations:** a College of Life Science, Liaoning Normal University, Dalian 116081, China; b Lamprey Research Center, Liaoning Normal University, Dalian 116081, China

**Keywords:** Lamprey, LECT2, Cell migration, Phagocytosis

## Abstract

The data presented in this article are related to the research article entitled “Characterization of the LECT2 gene and its protective effects against microbial infection via large lymphocytes in *Lampetra japonica*” (Wang et al., 2017) [1]. Here, we presented new original data about the effect of rL-LECT2 on cancer cells migration and macrophages phagocytosis. Wound healing assay and transwell chemotaxis assays were used to measure rL-LECT2 inhibition rates on cancer cell migration. Additionally, fluospheres beads and *Escherichia coli–*FITC were used to measure whether the rL-LECT2 can affect the phagocytosis of RAW264.7 cells.

**Specifications Table**

**Table:** 

Subject area	Biology
More specific subject area	Biochemistry, Genetics and Molecular Biology
Type of data	Figures
How data was acquired	Camera, Flow cytometry
Data format	Analyzed
Experimental factors	rL-LECT2 was added in Hela, MCF, RAW264.7 were incubated at 37 °C and 5% CO_2_.
Experimental features	Cell culture, wound-healing assays, transwell, phagocytosis
Data source location	Dalian, China
Data accessibility	The data are available with this article.

**Value of the data**

• A prokaryotic expression vector of lamprey LECT2 was constructed and rL-LECT2 protein was purified.
• The data provide information on the effect of rL-LECT2 on the migration of tumor cells rL-LECT2 treatment ca.
• The data provide information on the effect of rL-LECT2 on phagocytosis and inflammatory responses of RAW264.7 cells.

## 1 Data

**Fig. 1:**
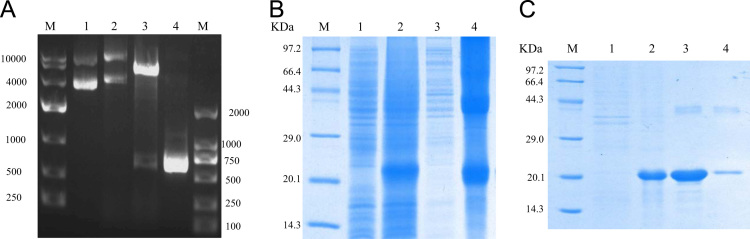
Cloning and Purification of rL-LECT2. (A) Construction of the L-LECT2-pCold Ι recombinant vector. M: DL 10,000 Marker; Lane1: pCold Ι plasmid product; Lane2: L-LECT2-pCold Ι recombinant plasmid; Lane3: L-LECT2-pCold Ι recombinant plasmid double enzyme digest; Lane 4: Colony PCR product; M: DL 2000 Marker. (B) SDS-PAGE analysis of the rLECT2 protein expressed in *Rosetta blue* bacteria. M: low molecular weight protein marker; Lane 1: non-induced expression of *Rosetta blue*/pColdI-rL-LECT2; Lane2: induced expression of *Rosetta blue*/pColdI-rL-LECT2; Lane3: supernatant; Lane4: inclusion body. (C) Purification of recombinant protein. M: low molecular weight protein marker; Lane1: flow through sample; Lane 2: 50 mM imidazole elute; Lane 3: 70 mM imidazole elute; Lane 4: 400 mM imidazole elute.

**Fig. 2:**
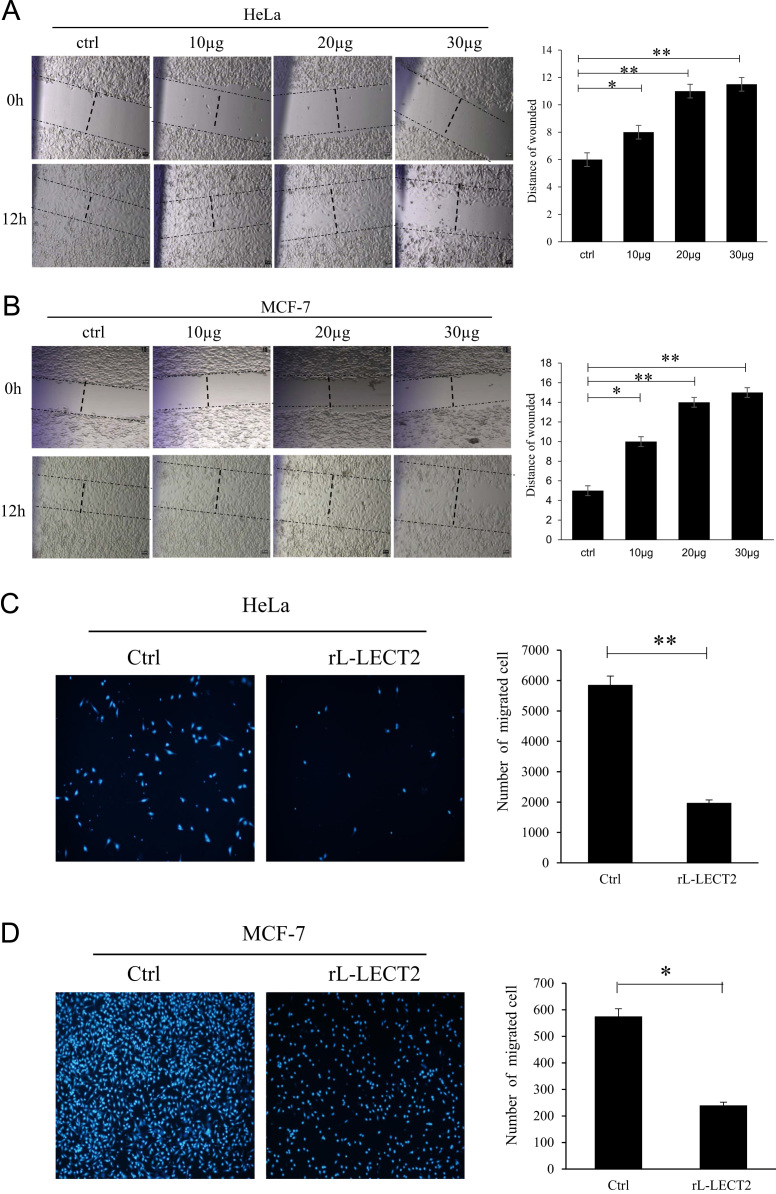
rL-LECT2 inhibits HeLa and MCF-7 cell migration. (A) rL-LECT2 remarkably inhibited HeLa cell migration in the wound healing assay. (B) rL-LECT2 remarkably inhibited MCF-7 cell migration in the wound healing assay. (C) rL-LECT2 inhibited HeLa cell migration in the Transwell assay. (D) rL-LECT2 inhibited MCF-7 cell migration in the Transwell assay. The migrated cell numbers of each condition were normalized to the negative control and quantified as the migration index. The error bars represent the means±SD (*p < 0.05; **p < 0.01).

**Fig. 3:**
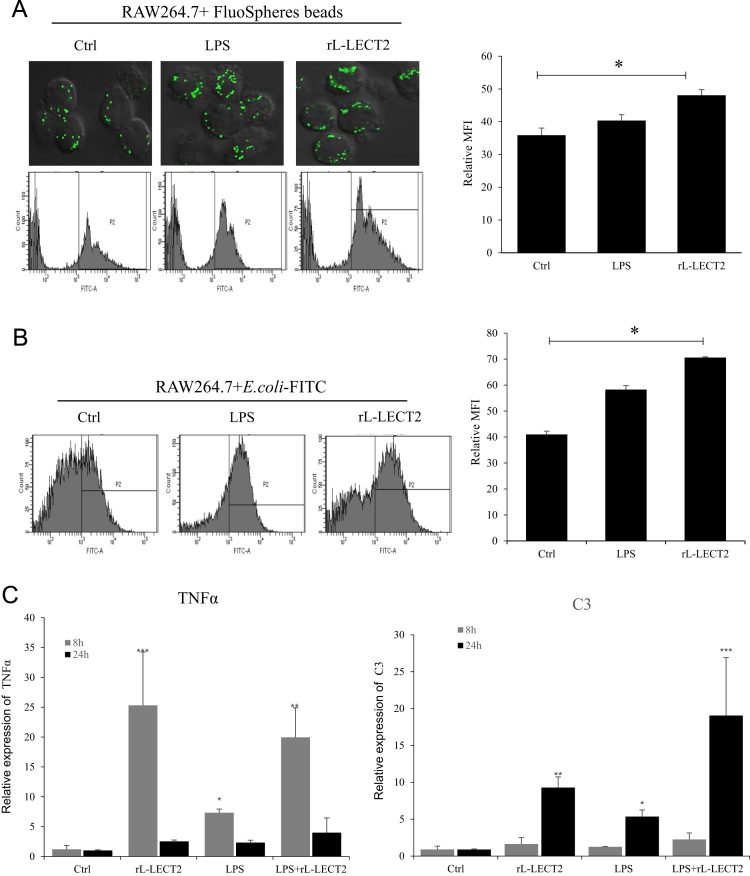
Effects of rL-LECT2 on the phagocytosis of mouse RAW264.7 cells. (A) Uptake of fluospheres beads with rL-LECT2 treatment. The phagocytosis of RAW264.7 cells was imaged by confocal microscopic analysis(upper pane). FACS analysis of phagocytizing fluospheres bead(low pane). (B) Uptake of *E. coli*-FITC with rL-LECT2 treatment. (C) rL-LECT2 can increase the transcript levels of the TNF-α and the complement molecular C3. RAW264.7 cells were incubated with rL-LECT2 for the indicated times (8 h and 24 h). The incubated cells were analyzed by qPCR to determine the transcript levels of TNF-α and C3. Each experiment was performed in triplicate. The error bars represent the means±SD (*p < 0.05;**p < 0.01).

The dataset of this article presented new original data about the effect of lamprey LECT2 on cancer cells migration and macrophages phagocytosis. Fig. 1, Fig. 2, Fig. 3 show the lamprey LECT2 can inhibit tumor cells migration and promote RAW264.7 cells phagocytosis.

## 2 Experimental design, materials and methods

### 2.1 Wound healing assay

MCF-7 and HeLa cells (5 × 10^4^ cells per well) were grown in 48-well plates overnight. Cells were wounded by scratching with pipette tips and washed with PBS. rL-LECT2 protein was added into the 48-well culture dishes. Images of the cells were taken using an inverted microscope (TE2000, Nikon, Japan) at 40× magnification after 12 h incubation. The cell migration from the edge of the injured monolayer was quantified by measuring the distance from the wound edges [1]. Cells receiving only medium served as a negative control. Each experiment was performed in triplicate.

### 2.2 Transwell migration assay

A modified Boyden chamber method using a 8-μm pore size polyvinylpyrrolidone-free polycarbonate membrane was carried out to assess the chemotactic activity of rL-LECT2 on leukocytes [2], [3]. During this assay, the MCF-7 cell or Hela cells were placed on the upper layer of a cell permeable membrane, and a solution containing either rL-LECT2, LPS or PBS was placed underneath the cell permeable membrane. Following an incubation period of 12 h. The nonmigrated cells on the upper side of the tissue culture plate insert were removed with a cotton swab and the migrated cells attached to the lower side of the membrane were stained with DAPI and counted using the High Content Imaging System (PerkinElmer). Each migration assay was performed in triplicate.

### 2.3 Measurement of phagocytosis

RAW264.7 cells were incubated with fluospheres beads or *E.coli*-FITC at a multiplicity of infection of 20 µg of rL-LECT2. Fluospheres beads and *E.coli*-FITC were used to investigate effect on phagocytosis of macrophage by flow cytometry and confocal microscopy. PBS and LPS were used as negative and positive controls, respectively. The assay was independently repeated three times.

## References

[1] Wang Z., Lu J., Li C., Li Q., Pang Y. (2017). Characterization of the LECT2 gene and its protective effects against microbial infection via large lymphocytes in Lampetra japonica. Dev. Comp. Immunol..

[2] Chen Q., Zhang M., Li Y., Xu D., Wang Y., Song A., Zhu B., Huang Y., Zheng J.C. (2015). CXCR7 mediates neural progenitor cells migration to CXCL12 independent of CXCR4. Stem Cells.

[3] Li T., Liu X., Shen Q., Yang W., Huo Z., Liu Q., Jiao H., Chen J. (2016). Salinomycin exerts anti-angiogenic and anti-tumorigenic activities by inhibiting vascular endothelial growth factor receptor 2-mediated angiogenesis. Oncotarget.

