# Supplementary material for: Data on functional characterization of LECT2 from Lampetra japonica
Source: Data Brief. 2018 Feb 15;17:1271–5. doi: 10.1016/j.dib.2018.02.036 (PMC5966521; doi:10.1016/j.dib.2018.02.036)
Supplement: Supplementary file 1 — Supplementary material [file mmc1.docx]

***Competing interests***

The authors declare that they have no competing interests.
